# ﻿A remarkable new species of the genus *Psammoecus* Latreille (Coleoptera, Silvanidae) from Lord Howe Island, Australia

**DOI:** 10.3897/zookeys.1161.100872

**Published:** 2023-05-11

**Authors:** Takahiro Yoshida, Chris A. M. Reid

**Affiliations:** 1 Systematic Zoology Laboratory, Department of Biological Sciences, Graduate School of Science, Tokyo Metropolitan University, 1-1 Minami-osawa, Hachioji, Tokyo, 192-0397 Japan Tokyo Metropolitan University Hachioji Japan; 2 Entomology, Australian Museum Research Institute, Australian Museum, 1 William Street, Sydney NSW 2010, Australia Australian Museum Sydney Australia

**Keywords:** Brachyptery, endemism, southwest Pacific, taxonomy, Telephanini

## Abstract

A new species, *Psammoecuslordhowensis***sp. nov.**, is described from Lord Howe Island, Australia. The new species is brachypterous and most likely endemic to the island. This species is distinct and can be distinguished by the following morphological characters: body rounded and convex; eyes small; temples well developed; lateral pronotal teeth absent; and hind wing strongly reduced.

## ﻿Introduction

Lord Howe Island is a small volcanic island (1455 ha) situated in the temperate zone of the Tasman Sea, about 600 km from the east sea coast of Australia (Hutton et al. 2007). It was formed about 6.9 million years ago (McDougall et al. 1981). The native biota of this island is diverse with a high degree of endemism (Hutton et al. 2007). Several endemic genera are present on the island, for example the palms *Howea* Becc., *Hedyscepe* H. Wendl. & Drude and *Lepidorrhachis* (H. Wendl. & Drude) O. F. Cook, a woody composite *Lordhowea* B. Nord., the tree *Negria* F. Muell., the leech *Quantenobdella* Richardson, three annelid genera (*Paraplutellus* Jamieson, *Pericryptodrilus* Jamieson and *Eastoniella* Jamieson), an isopod *Stigmops* Lillemets & Wilson, a hemipteran bug *Howeria* Evans and a cricket *Howeta* Otte & Rentz (Hutton et al. 2007). The native flora includes 241 species, of which 43.6% are endemic (Green 1994). Although over 50% of the terrestrial invertebrates of Lord Howe are probably endemic species (Recher and Ponder 1981), many species still have not been described or recorded (Hutton et al. 2007).

Silvanidae are generally small, cryptic, detritivores, feeding on dead plant material and fungi in closed forests (Lawrence and Ślipiński 2013). The Australian fauna has not been revised, but is known to include about 75 species, many of which are undescribed (Lawrence and Ślipiński 2013). Two Silvanidae are recorded from Lord Howe, *Australodendrophagusaustralis* (Erichson) and the cosmopolitan species *Cryptamorphadesjardinsii* (Guérin-Menéville) (Olliff 1889). Here we describe a third, new species, in *Psammoecus* Latreille. *Psammoecus* includes 84 species and is characterized by the securiform distal maxillary palpomere, the simple scutellary shield, lack of a scutellary striole, and the bilobed third tarsomere (Thomas 1984; Thomas and Nearns 2008; McElrath et al. 2023). Although the distribution of most species is restricted to the Old World, two Old World *Psammoecus* species have been found in the New World: *P.trimaculatus* Motschulsky from Brazil and *P.simonis* Grouvelle from the airport of Minnesota, United States (Thomas and Yamamoto 2007; Ouellette 2018). Some *Psammoecus* species show an extremely wide distribution, which seems to be sometimes caused by human activity (Karner 2020) and several intercepted records at ports or airports with imported materials have been published (e.g., Lu and Han 2006; Ouellette 2018; Yoshida 2020). Seven *Psammoecus* species are known from Australia, three of which are endemic, but the others may be accidental introductions (Karner 2020). The distinctive new species described here is flightless and almost certainly endemic to Lord Howe Island.

## ﻿Material and methods

### ﻿Observation, dissection and photographic techniques

Observations and dissections were performed under a stereomicroscope (Olympus SZX10 or Nikon SMZ1270). Male genital structures were placed on a cavity slide glass with Euparal for observation under an optical microscope (Nikon Eclipse E400). Measurements were made using a digital microscope (Olympus DSX110) with an integrated measuring function and read up to three decimal places in millimeters.

The abdomens of some specimens were removed and soaked in a 10% potassium hydroxide solution at room temperature overnight. After rinsing in water, the soaked abdomen was dissected under a stereomicroscope (Nikon SMZ1270) using fine insect pins, and the genital organs were detached for observation. After observations were completed, the dissected parts were mounted in Euparal on cover glasses which were glued to a piece of cardboard, and pinned with the relevant specimen (Maruyama 2004).

Photographs were taken with a digital camera (Canon EOS 7D) fitted with a macro lens (Canon MP-E 65 mm). Composite images were produced using Affinity Photo version 1.10.6 (Serif Europe Ltd.). Images were retouched using the same software.

### ﻿Terminology, abbreviations and specimen deposition

Morphological terminology follows Lawrence et al. (2010) and Lawrence et al. (2011). Abbreviations and measurements are as follows:

**BL**HL + PL + EL;

**HL** length from anterior margin of clypeus to imaginary line between posterior margins of temples in dorsal view measured along the median line;

**HW** greatest width of head across eyes;

**IE** narrowest width of interspace between eyes;

**PL** length of pronotum measured along the median line;

**PW** greatest width of pronotum, excluding teeth;

**EL** length of elytra measured along suture plus length of scutellar shield;

**EW** greatest combined width of elytra.

Depositories of the examined specimens are as follows:

**ANIC**Australian National Insect Collection, CSIRO, Canberra, Australia;

**AM**Australian Museum, Sydney, Australia.

## ﻿Results

### ﻿Taxonomy

**Family Silvanidae Kirby, 1837**

**Subfamily Brontinae Erichson, 1845**

**Tribe Telephanini LeConte, 1861**

**Genus *Psammoecus* Latreille, 1829**

#### ﻿ Psammoecus lordhowensis

Taxon classificationAnimaliaColeopteraSilvanidae

Yoshida & Reid sp. nov.

17939FB8-EF7A-5A29-BB25-36D71CE71B5D

https://zoobank.org/F0ABF9A7-2F5D-4BC1-9506-9B19DEC19489

Figs 1 , 2

##### Diagnosis.

This new species is distinguished from other *Psammoecus* species by the rounded and convex body shape, small eyes, well-developed temples, the pronotum with irregular crenulation of obtuse tubercles (shorter than wide) not forming obvious teeth, and the extremely reduced hindwing and the male genital morphology.

##### Description.

**BL**: 2.79–3.50 mm (*n* = 20).

**Figure 1.**
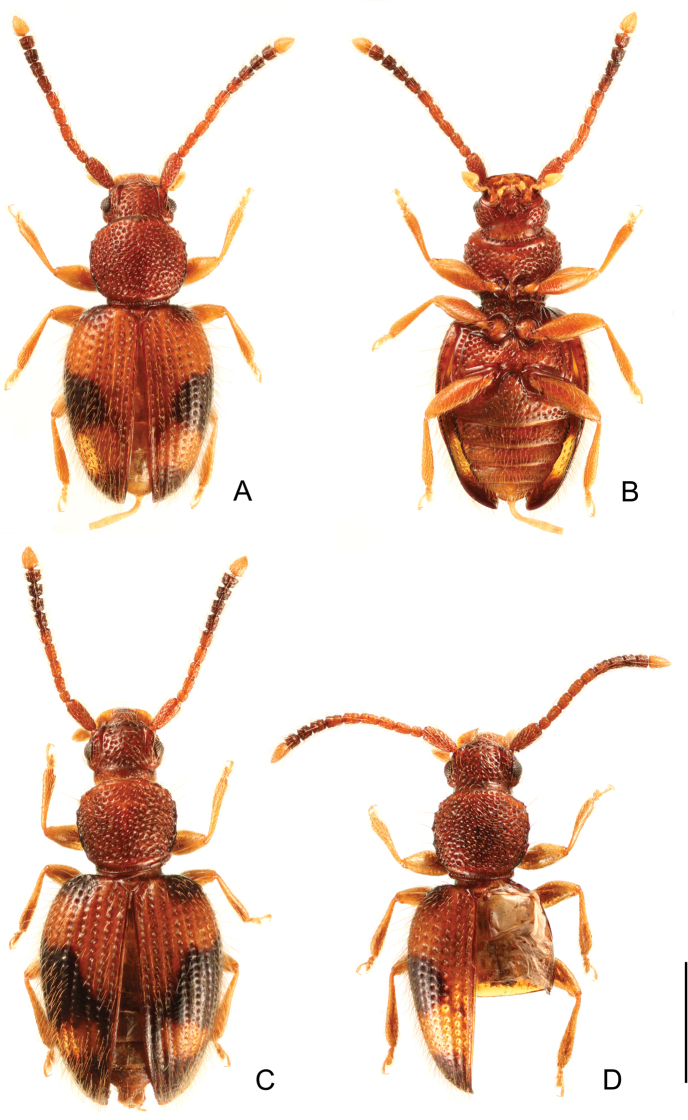
Habitus of *Psammoecuslordhowensis* sp. nov. **A, B** holotype (male), dorsal (**A**) and ventral views (**B**) **C** darker colored specimen, dorsal view **D** specimen of which right elytron and abdomen were removed, dorsal view. Scale bar: 1.0 mm.

***Coloration*** (Fig. 1). Head and pronotum reddish brown. Elytra reddish brown or somewhat lighter colored, with a quadrate or triangular black macula on each elytron at middle, narrowly darkened around humeri and apex of elytra; middle macula and apical darkened area connected by darkened area along lateral margin, also connected by darkened area along suture in darker colored specimens (Fig. 1C). Mouthparts and legs yellowish brown. Antennae reddish brown or somewhat lighter colored, 7^th^ antennomere dark brown, 8^th^ to 10^th^ black, 11^th^ white; setae golden.

***Head*** (Fig. 1). Subquadrate, longer than wide, HL: 0.47–0.61 mm, HW: 0.58–0.71 mm, HW/HL: 1.14–1.31; IE/HL 0.89–1.02 (*n* = 20); Temples well developed, narrowed immediately at base. Eyes small, round, laterally prominent. Punctation strong, moderately dense, without microsculpture on the interstices. Antennae very long; antennomeres with pubescence of moderate to large length; distal portion of 7^th^ to 10^th^ and entire 11^th^ antennomeres with short pubescence, denser on 9^th^ to 11^th^ antennomeres; with reticulate microsculpture on 2^nd^ to 10^th^ antennomeres; antennal total length and antennomere approximate length ratios from base to apex, both for the holotype, 1.68 mm; 3.6: 1.0: 1.5: 1.5: 1.5: 1.5: 1.6: 1.4: 1.3: 1.2: 2.2.

***Pronotum*** (Fig. 1). Subquadrate, widest near middle, with slightly enlarged margins, PL: 0.70–0.90 mm, PW: 0.56–0.86 mm, PL/PW: 0.91–1.29 (*n* = 20), without obvious lateral teeth. Punctation on pronotal disk as on vertex. Pubescence composed of setae of moderate length, very long setae on tubercles on lateral margins and anterior and posterior angles. Anterior angle with a few tiny setiferous tubercles; lateral margin with 2–4 obtuse setiferous tubercles (shorter than wide) of similar size, not acute teeth; distances between setiferous tubercles on lateral margins irregular; posterior angle with a long seta.

***Legs*** (Fig. 1) moderate length, without microsculpture; femora thick; tibiae somewhat thick, gradually widening distally; tarsi moderate length.

***Elytra*** (Fig. 1). Oval, EL: 1.60–2.02 mm, EW: 1.08–1.47 mm (greatest width at anterior 1/3), EW/BL: 0.38–0.44, EW/EL: 0.67–0.74 (*n* = 20), convex, with poorly developed humeri, with moderately rounded apices. Strial punctures moderate depth, mostly same width of the interstices. Pubescence composed of numerous semi-erect setae of medium length, erect long setae along margins and humeri, gradually shorter toward apices.

***Scutellary shield*** triangular, with several short setae.

***Hindwing*** (Fig. 1D) strongly reduced (brachypterous), about 1/4× as long as elytron, lacking venation.

**Figure 2.**
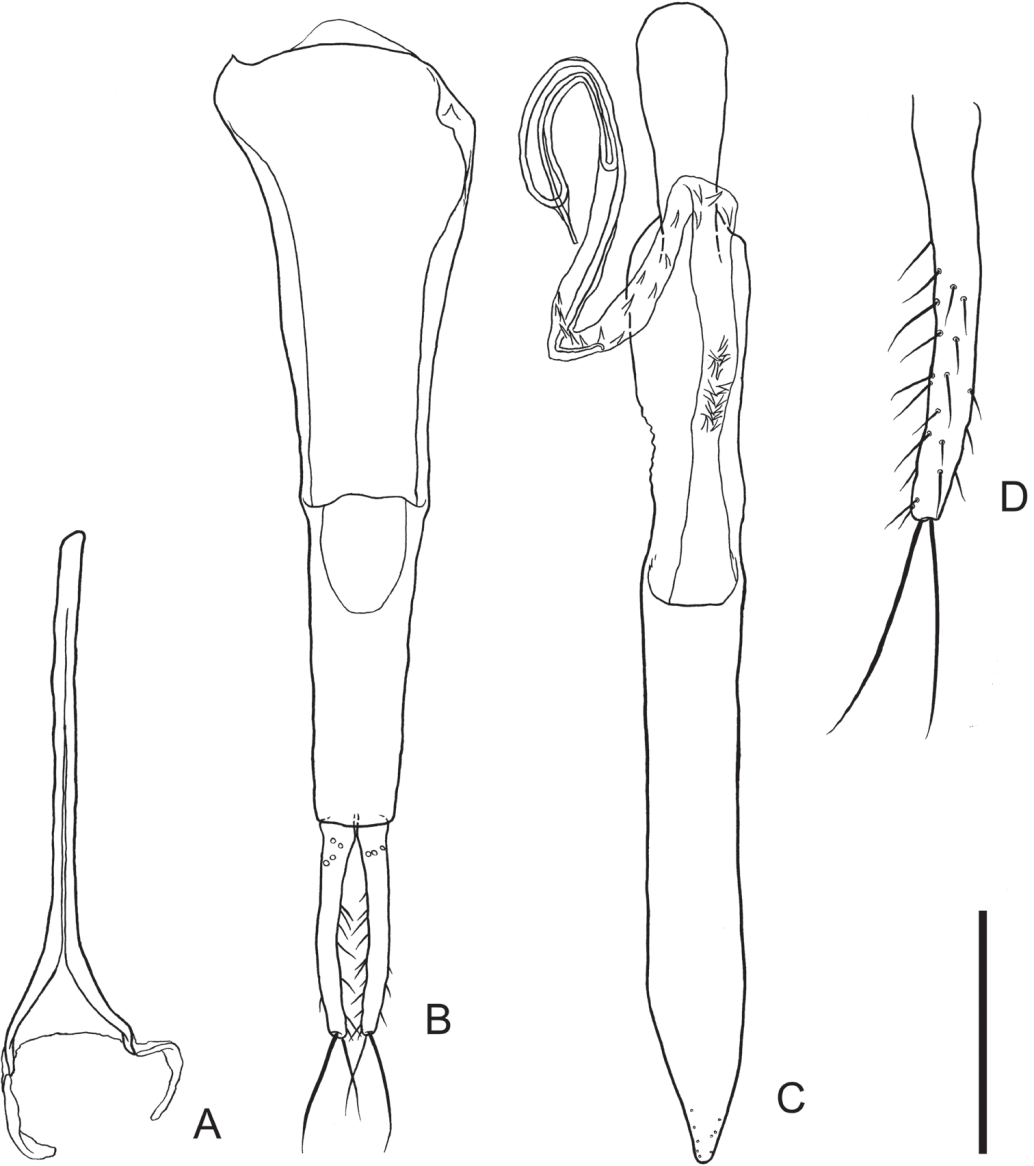
Male genital organs of *Psammoecuslordhowensis* sp. nov. **A** spiculum gastrale, ventral view **B** phallobase, ventral view **C** penis, dorsal view **D** right paramere, dorsal view. Scale bars: 0.1 mm (**A–C**); 0.2 mm (**D**).

***Male genitalia*** (Fig. 2). Spiculum gastrale (Fig. 2A) with elongate strut, moderately diverging around posterior 2/9; posterior half of branches slightly narrowed, curved inwards near apices, connected by a membrane; lateral sclerites membranous, elongate, curved inwards. Parameres (Fig. 2B, D) stick-shaped, elongate, sub-parallel, slightly curved inwards, dorsally with a few punctures near bases, ventrally with setae of various lengths on posterior 3/4 (Fig. 2D), dorsally without setae, with two very long setae on each apex. Phallobase (Fig. 2B) elongate, sub-parallel; tegminal strut longer than basal piece; basal piece slightly narrowed toward posterior, with anterior margin widely incised at anterior 1/3. Penis (Fig. 2C) elongate, sub-parallel, apical 1/6 narrowed, roundly protruded at apex, with some fine punctures near apex. Internal sac moderate length, recurved around apex, apically with a thin ringed structure and a thin long apical strut, sparsely armed with some spines on middle area, densely with spines near basal piece.

##### Type series.

***Holotype***: male, ‘NSW; On walking track to Erskine | Valley, adjacent to Salmon Beach, Lord | Howe Island; -31:33:39; 159:4:31; 10- | Dec-2000; G. Cassis; LHI/GC/L18 leaf | litter ex collected at night’, ‘+3 in vial’, ‘K188166’ (AM). ***Paratypes*** (33 specimens): [Lord Howe Island] • 1 male; Anderson Road, south end; 40 m; 16 Nov. 1979; G. B. Monteith; Calcareous soil, sieved litter; Q. M. Berlesate No. 149, Pickard Veg: DaCt [*Drypetesaustrasica*-*Cryptocaryatriplinervis*] (ANIC: male genital structures illustrated and photo presenting brachyptrery, Figs 1D and 2) • 1 male; same label data (ANIC) • 1 female; summit of Dawson Point Ridge; 150 m; 5 Nov. 1979; G. B. Monteith.; volcanic soil, sieved litter; Q. M. Berlesate No. 120; Pickard Veg: DaCt; voucher Specimen 81–106 (ANIC) • 1 ex.; same locality; 7 Nov. 1979; G. B. Monteith; same microhabitat and collecting method; Q. M. Berlesate No. 128, Pickard Veg: DaCt (ANIC) • 1 ex.; eastern slope of Dawsons Point Ridge above old settlement; 31°31'15"S, 159°3'7"E; 1 Dec. 2000; CBCR, Australian Museum; Leaf litter ex Closed Rain Forest *Drypetes*/*Cryptocarya* (exposed) habitat; LHIS014L, K188156 (+5) (AM) • 1 ex.; Intermediate Hill, near summit; 250 m; 6 Nov. 1979; G. B. Monteith; volcanic soil, sieved litter; Q. M. Berlesate No. 123, Pickard Veg: DaCt (ANIC) • 1 ex.; 100 m into Intermediate Hill from Kings Beach side; 31°33'S, 159°05'E; 8 Dec. 2000; C. Reid; palm leaf litter; K188160 (AM) • 1 male; Malabar Ridge saddle; 120 m; 25 Nov. 1979; G. B. Monteith; volcanic soil, sieved litter; Q. M. Berlesate No. 167, Pickard Veg: DaCt (ANIC) • 1 ex.; western slope of Malabar Ridge S of Kims Lookout trail; 31°30'57"S, 159°3'31"E; 24 Nov. 2000; CBCR, Australian Museum; leaf litter ex Broad Megaphyllous Closed Sclerophyll Forest – *Howeabelmoryana* habitat; LHIS007L, K188158 (+1 in vial) (AM) • 1 ex.; on walking track to Erskine Valley, adjacent to Salmon Beach; 31°33'39"S, 159°4'31"E; 10 Dec. 2000; G. Cassis; leaf litter ex collected at night; LHI/GC/L18, K188157 (+1 in vial) (AM) • 1 female: North Bay trail, 50 m from junction with Kims Lookout trail; 31°31'4"S, 159°3'0"E; 11 Dec. 2000; G. Cassis; leaf litter ex; LHI/GC/L22, K188165 (AM) • 2 exs; Stevens Reserve; 10 m; 13 Nov. 1979; G. B. Monteith; calcareous soil, sieved litter; Q. M. Berlesate No. 144, Pickard Veg: DaCt (ANIC) • 1 ex.; Stephens Reserve; 31°31'15"S, 159°03'53"E; 13 Dec. 2000; CBCR; leaf litter; LHIS059L, K188155 (AM) • 1 female and 1 ex.; Stephens Reserve, c. 10 m; 31°31'33"S, 159°03'53"E; 9 Dec. 2000; C. Reid; Palm frond leaf litter; K188161(188162) (AM) • 1 ex.; junction of Kims Lookout trail and North Beach trail; 31°31'8"S, 159°3'0"E; 18–27 Feb. 2001; Australian Museum; pit trap; LHIS010/05, K188163 (AM) • 1 female; “Little Slope“; 31°35'12"S, 159°4'3"E; 30 Nov. 2000; CBCR, Australian Museum; leaf litter ex Broad Megaphyllous Closed Sclerophyll Forest – *Howeabelmoryana* habitat; LHIS051L, K188154 (+1) (AM) • 1 ex.; “Get Up Place“, trail to Mt. Gower; 31°34'58"S, 159°4'52"E; 2 Dec. 2000; CBCR, Australian Museum; Leaf litter ex Broad Closed Sclerophyll Scrub, *Dracophyllum*/*Metrosideros* habitat; LHIS048L, K188159 (AM) • 1 ex.; Mt Gower tk [track]; 31°34'43"S, 159°05'6"E; 330 m; 10–17 May 2004; N. Velez; Site G13 litter *Chion.quadristamineus* forest; Australian Museum K517792 (AM) • 1 ex.; same locality, geo-coodinate and altitude; 1–12 Nov. 2005; N. Velez; same microhabitat; Australian Museum K517794 (AM) • 1 ex.; Mt Gower tk; 31°34'44"S, 159°05'7"E; 360 m; 10–17 May 2004; N. Velez; Site G14 litter *Chion.quadristamineus* forest; Australian Museum K517793 (AM) • 1 ex.; Mt Gower tk; 31°34'22"S, 159°04'42"E; 160 m; 10–17 May 2004; N. Velez; Site G6 litter *Syzigiumfullagarii* forest; Australian Museum K517791 (AM) • 1 ex.; Mt Gower tk; 31°34'41"S, 159°05'3"E; 260 m; 1–12 Nov. 2005; N. Velez; Site G10 litter *Chion.quadristamineus* forest; Australian Museum K517795 (AM) • 1 ex.; Mt Gower tk; 31°34'49"S, 159°05'9"E; 460 m; 10–17 May 2004; N. Velez; Site G18 litter *Chion.quadristamineus* forest; Australian Museum K517796 (AM) • 1 male; Mt Gower tk; 31°34'45"S, 159°05'7"E; 390 m; 10–17 May 2004; N. Velez; Site G15 litter *Chion.quadristamineus* forest; Australian Museum K517797 (AM) • 1 female; Mt Gower tk; 31°34'52"S, 159°05'8"E; 530 m; 1–12 Nov. 2005; N. Velez; Site G21 litter *Draco Metrosiderosnervulosa* scrub; Australian Museum K517798 (AM: habitus image taken, Fig. 1C) • 1 ex.; Mt Gower tk; 31°34'43"S, 159°05'4"E; 290 m; 1–14 Apr. 2006; N. Velez; Site G11 litter *Chion.quadristamineus* forest; Australian Museum K517799 (AM) • 1 female; Forest behind Salmon Beach; 31°33'96.2"S, 159°04'33.3"E; 29 m; 10 Feb. 2017; Jenkins Shaw & Jensen; under bark, fallen tree; LHI2017Feb10-J23, Australian Museum K517790 (AM) • 1 female; SEern [southeastern] face of Mt Lidgberg, at base of summit tabletop; 31°34'26"S, 159°4'54"E; 2–12 Dec. 2000; CBCR, Australian Museum; Pit trap; LHIS031/05; K188164 (AM) • 2 exs; Mt Lidgbird E shelf; 31°33'82.6"S, 159°05'27.1"E; 486 m; 9 Feb. 2017; Jenkins Shaw & Jensen; shifting leaf litter; LHI2017Feb9-J20a, Australian Museum K517801 (517802) (AM) • 1 ex.; Mt Lidgbird tk; 31°33'44"S, 159°05'38"E; 390 m; 10–27 May 2004; N. Velez; Site L12 litter *Drypetes*/*Cryptocarya*; Australian Museum K517800 (AM).

##### Distribution.

Lord Howe Island (New South Wales, Australia).

##### Habitat.

*Psammoecuslordhowensis* is endemic to the Lord Howe main island, where it is widespread in closed temperate rainforest, from the northern (Malabar Ridge) to the southern (Little Slope) end of the island, and from sea level to 530 m elevation. It does not appear to occur in the cloud forest on the summit of Mount Gower (above 700 m). This species is mostly collected by sieving leaf litter, but one specimen was collected in a pitfall trap and another under bark.

##### Etymology.

The specific name of this new species is derived from the type locality, as a noun in genitive case.

## ﻿Discussion

### ﻿Hindwing reduction in the tribe Telephanini

The hindwing of this new species is extremely reduced (brachypterous), which means that it cannot fly. In *Psammoecus*, brachypterous or apterous species have not been recorded previously. In related genera, four apterous species are known in *Telephanus* from Jamaica (2 spp.), Reunion Island (1 sp.) and Mexico (1 sp.), and one brachypterous species is known in *Cryptamorpha* (*C.triregia*) from the Three Kings Islands, New Zealand (Thomas 1984, 1992, 2011; Brown et al. 2012). Additionally, several flightless species of Telephanini are known from rainforests on the Australian mainland (Lawrence and Ślipiński 2013). The loss of the ability to fly among animals is characteristic of the well-known “island syndrome” (Carlquist 1974; Baeckens and Van Damme 2020). Although, in general, many species of *Psammoecus* have high-flight activity (Karner 2020), this new species has likely reduced its flight activity and lost its functional hindwing in Lord Howe Island.

### ﻿Which species is most closely related to this new species?

In *Psammoecus*, the male genital structures (including the spiculum gastrale and the internal sac, which are sometimes overlooked in descriptions) are similar to each other between closely related species (e.g., *P.trimaculatus*, *P.triguttatus* Reitter and *P.labyrinthicus* Yoshida & Hirowatari; *P.fasciatus* Reitter and *P.hiranoi* Yoshida & Hirowatari) (Yoshida and Hirowatari 2013, 2014; Yoshida et al. 2018; Karner 2020). For identification of these species, it is often necessary to examine male genital morphology. The male genital structure of this new species is similar to those of some *Psammoecus* species (e.g., *P.venustus* Karner, *P.taiwanensis* Yoshida, Karner & Hirowatari). However, due to its distinctive morphology (see Diagnosis), this new species can be distinguished from other congeneric species without examination of its male genital structure. In addition, information on the whole male genital morphology of other *Psammoecus* species is significantly lacking. Although some species have similar male genitalia to those of this new species, it is difficult to conclude which species is closely related to this new species. To determine the species most closely related to this new species, further studies on the male genital morphology of this genus and the phylogenetic relationships among this genus are required.

## Supplementary Material

XML Treatment for Psammoecus lordhowensis
